# Supplementary material for: FIB-4 and APRI as Predictive Factors for Short- and Long-Term Survival in Patients with Transjugular Intrahepatic Portosystemic Stent Shunts
Source: Biomedicines. 2022 Apr 28;10(5):1018. doi: 10.3390/biomedicines10051018 (PMC9138812; doi:10.3390/biomedicines10051018)
Supplement: Supplementary file 1 [file biomedicines-10-01018-s001.zip › biomedicines-1608155-supplementary.pdf]

biomedicines

MDPI

Table S1. Baseline demographic and clinical characteristics of the validation co-hort.

	**All patients**
Patients, n (%)	187 (100)
Age, y	59 (52-67)
Male/female, n (%)	115/72 (62/38)
Etiology of cirrhosis	
Alcohol/viral/autoimmune/NASH/other, n	112/16/2/18/49
Child Pugh A/B/C, n	6/163/18
MELD	13 (10-16)
Previous SPB yes/no, n (%)	77/110 (41/59)
HE, n (%)	8 (4)
TIPS procedural characteristics	
Pre-TIPS PSG, mmHg	15 (12-18)
Post-TIPS PSG, mmHg	5 (4-7)
Reduction PSG, mmHg	10 (7-13)
Stent diameter, mm	8 (8-8)
Laboratory characteristics	
Leukocytes, /nl	5.9 (4.4-8.5)
Hemoglobin, g/dl	10 (8.8-11.7)
Platelets, /nl	126 (87-181)
INR	1.25 (1.15-1.41)
Sodium, mmol/l	135 (131-138)
Creatinine, mg/dl	1.3 (0.9-1.7)
Bilirubin, mg/dl	1.0 (0.6-1.5)
AST, U/l	42 (32-55)
ALT, U/l	23 (15-34)
Albumin, g/dl	2.8 (2.4-3.2)
CRP, mg/dl	1.1 (0.5-2.0)

Abbreviations: NASH: non-alcoholic steatohepatitis; MELD: model of end-stage liver disease; SPB: Spontaneous bacterial peritonitis; HE: hepatic encephalopathy, TIPS: transjugular portosystemic shunt insertion; PSG: portosystemic pressure gradient; INR: international normalized ratio; AST: aspartate aminotransferase; ALT: alanine aminotransferase; CRP: C-reactive protein; Shown is median with IQR or numbers with percentages.

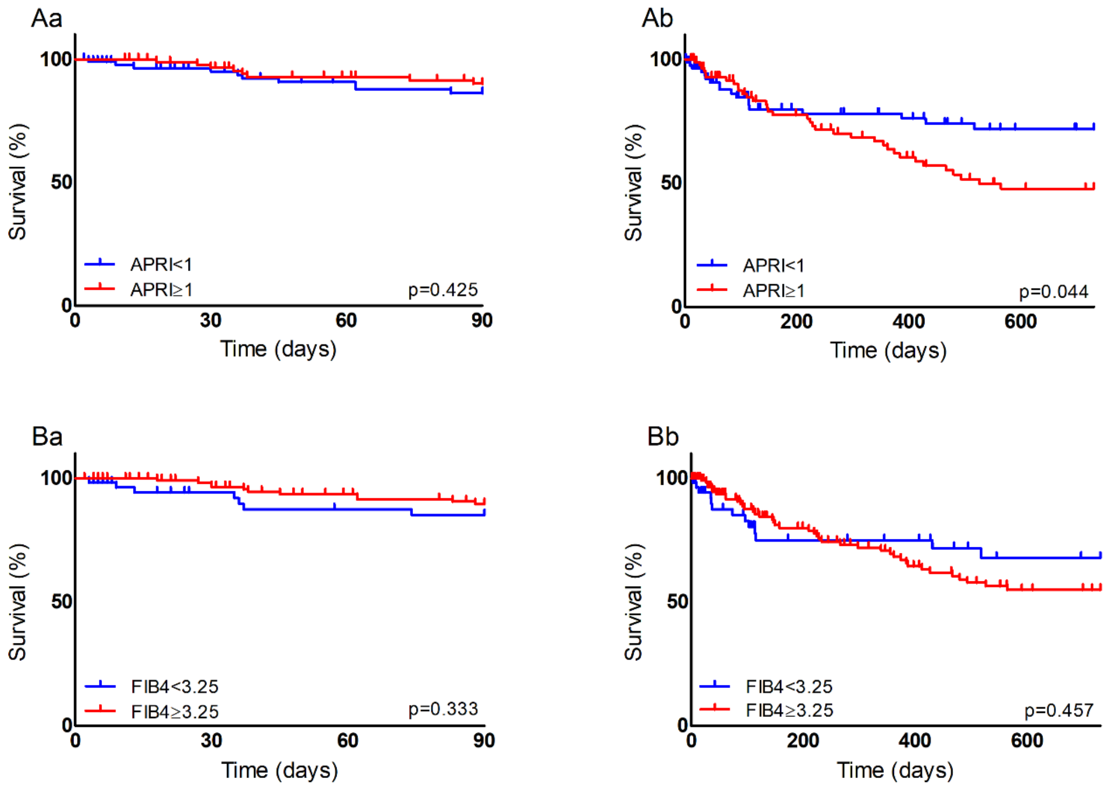

Figure S1. 90-days (a) and 730 days (b) LTF survival of TIPS patients in the valida-tion cohort in dependence of A) APRI (<1 vs. ≥1) and B) FIB-4 (<3.25 vs. ≥3.25), p-values were ob-tained using the log-rank test.

Table S2. Multivariate Cox-regression analyzing risk factors for liver-related event incidence after TIPS insertion. All significant parameters tested in the univariate analysis were in-cluded in the multivariate model.

**Risk factor for event**	**Multivariate analysis 24 months**		
	HR	SE	p
MELD	1.140	.051	.010
FIB-4	1.267	.081	.003

Abbreviations: MELD: model of end-stage liver disease; FIB-4: Fibrosis-4 score; HR: hazard ratio; SE: standard error.
